# Assessing the relationship between levator palpebrae superioris and thyroid-associated ophthalmopathy using the Dixon-T2WI sequence

**DOI:** 10.3389/fendo.2024.1387217

**Published:** 2024-05-28

**Authors:** Dan Liu, Yongbo Duan, Kai Huang, Cheng Song, Yufeng Ouyang, Xiaoxin Lin, Jie Shen, Haixiong Chen

**Affiliations:** ^1^ Department of Radiology, Shunde Hospital, Southern Medical University, Foshan, China; ^2^ Department of Ophthalmology, Shunde Hospital, Southern Medical University, Foshan, China; ^3^ Department of Endocrinology and Metabolism, Shunde Hospital, Southern Medical University, Foshan, China

**Keywords:** thyroid-associated ophthalmopathy, levator palpebrae superioris muscle, Dixon-T2WI magnetic resonance imaging, condition assessment, quantitative evaluation

## Abstract

**Background:**

The current clinical practice lacks sufficient objective indicators for evaluating thyroid-associated ophthalmopathy (TAO). This study aims to quantitatively assess TAO by evaluating levator palpebrae superioris (LPS) using Dixon-T2WI.

**Methods:**

The retrospective study included 231 eyes (119 patients) in the TAO group and 78 eyes (39 volunteers) in the normal group. Dixon-T2WI provided data on maximum thickness of LPS (LPS_T) and signal intensity ratio (LPS_SIR) between the muscle and ipsilateral brain white matter. TAO diagnosis and assessment of its activity and severity were quantitatively determined using LPS_T and LPS_SIR.

**Results:**

In the TAO group, LPS_T and LPS_SIR were higher than those in the normal group (*p* < 2.2e-16). The upper lid retraction (ULR) ≥ 2 mm group exhibited higher LPS_T and LPS_SIR compared to the ULR < 2 mm and normal groups. Optimal diagnostic performance was achieved with an AUC of 0.91 for LPS_T (cutoff: 1.505 mm) and 0.81 for LPS_SIR (cutoff: 1.170). LPS_T (*p* = 2.8e-07) and LPS_SIR (*p* = 3.9e-12) in the active phase were higher than in the inactive phase. LPS_T and LPS_SIR showed differences among the mild, moderate-to-severe, and sight-threatening groups (*p* < 0.05). ROC showed an AUC of 0.70 for LPS_T (cutoff: 2.095 mm) in judging the active phase, and 0.78 for LPS_SIR (cutoff: 1.129). For judging the moderate-to-severe and above, AUC was 0.76 for LPS_T (cutoff: 2.095 mm) and 0.78 for LPS_SIR (cutoff: 1.197).

**Conclusion:**

The maximum thickness and SIR of LPS provide imaging indicators for assisting in the diagnosis and quantitative evaluation of TAO.

## Introduction

Thyroid-associated ophthalmopathy (TAO) is an autoimmune disease associated with thyroid disease, with symptoms that may include upper lid retraction (ULR), diplopia, and vision loss, seriously affecting patients’ quality of life (1–5). According to the latest guidelines for TAO management (6, 7), the optimal treatment strategy primarily relies on the Clinical Activity Score (CAS) and the European Group on Graves’ orbitopathy (EUGOGO) classification of severity. However, the subjective nature and limited comparability of CAS and the EUGOGO classification of severity pose challenges in accurately reflecting the size, morphology, and internal changes of orbital tissue (6). Consequently, the objective assessment of TAO becomes a challenging task, emphasizing the crucial role of objective indicators in evaluating TAO.

ULR stands out as a pivotal diagnostic criterion and common ocular manifestation in TAO, serving as an important indicator for assessing the disease’s severity (8, 9). Previous investigation indicates that immune-reactive inflammation of the levator palpebrae superioris (LPS) contributes significantly to ULR (10, 11). Therefore, gaining a comprehensive understanding of LPS characteristics and its relationship with disease progression is essential for the objective evaluation of TAO. Direct observation of LPS in clinical practice is challenging. Magnetic resonance imaging (MRI) is a fundamental diagnostic tool utilized in clinical settings of TAO, providing visualization of orbital tissues (including LPS) and monitoring dynamic changes in orbital tissues (12–16). Despite numerous studies using MRI in TAO, there are still considerable debates about the evaluative capabilities of various MRI techniques in TAO (17, 18). Our previous research indicates that the Dixon technique has significant advantages in assessing TAO (19).

The Dixon-T2WI sequence is a fat-suppression technique based on chemical shift analysis, allowing effective separation of water and fat (18–20). Compared to inversion recovery-based fat-suppression sequences, Dixon-T2WI is less susceptible to magnetic susceptibility artifact (21). The short acquisition duration is attributed to a high chemical shift at high field (22). These advantages make Dixon-T2WI well-suited for head, neck, and orbital imaging (23), providing a more precise visualization of subtle inflammatory changes within LPS (24). Previous studies have suggested the utility of measuring the maximum thickness of LPS (LPS_T) and signal intensity ratio (LPS_SIR) between the muscle and ipsilateral brain white matter in MRI sequences as crucial indicators for evaluating TAO (25, 26). However, limited research has employed Dixon-T2WI technology for quantitative assessment of its activity and severity, providing numerical cutoff points.

Our study utilized Dixon-T2WI to investigate the relationship between LPS and ULR, CAS, and the EUGOGO classification of severity in TAO, aiming to assess the diagnostic value of LPS in TAO and evaluate its condition.

## Materials and methods

This study obtained approval from the Institutional Review Board (IRB: KYLS20220723), and informed consent was obtained from all participants. The study focused on a single orbit. Retrospective data were collected from 280 patients who underwent orbital MRI at our hospital from October 2020 to November 2023. The indications for orbital MRI in patients are as follows: (a) when clinicians initially suspect a patient has TAO, performing orbital MRI helps distinguish it from other orbital diseases; (b) when clinicians have made a provisional diagnosis of TAO in a patient, conducting orbital MRI helps determine the detailed severity and extent of the disease; (c) when clinicians have confirmed a diagnosis of TAO in a patient, performing orbital MRI is used to regularly monitor the progression of the disease; and (d) orbital MRI are performed on TAO patients after treatment to evaluate treatment efficacy. The inclusion criteria for affected eyes in the TAO group were as follows: (a) meet the diagnostic criteria for TAO according to Bartley’s criteria (8), and (b) complete clinical data or MRI image data. The exclusion criteria for affected eyes in the TAO group were as follows: (a) history of previous eye surgery or trauma; (b) prior history of orbital radiation therapy; and (c) large MRI image artifacts and poor image quality. A total of 329 eyes were excluded based on the inclusion and exclusion criteria, leaving 231 eyes (119 patients) in the TAO group. Concurrently, 78 eyes (39 volunteers) were recruited as the normal group. The inclusion criteria for the normal group were as follows: (a) the absence of thyroid eye disease and other orbital diseases; (b) no history of previous eye surgery or trauma; and (c) complete MRI data and good image quality (**Supplementary Figure S1**). Subjects underwent orbital examination with a 3-Tesla Skyra MRI System (Siemens Healthcare, Erlangen, Germany) within 7 days of the ophthalmic examination. Clinical information of the subjects was collected, including age, gender, smoking history, duration of TAO, thyroid-stimulating hormone (TSH), free thyroxine (FT4), thyrotropin receptor-stimulating antibody (TSAb), ULR, diplopia, treatment, exophthalmos by Hertel, CAS, the EUGOGO classification of severity, and the degree of ULR. Subject demographics are shown in **Table 1**.

**Table 1:** General characteristics of subjects.

	Normal group	CAS group		EUGOGO group		
		Inactive phase	Active phase	Mild	Moderate-to-severe	Sight-threatening
Eyes, n (%)	78 (100.00)	81 (35.06)	150 (64.94)	79 (34.20)	147 (63.64)	5 (2.16)
Male, n (%)	38 (48.72)	32 (39.51)	89 (59.33)	30 (37.97)	86 (58.53)	5 (100.00)
Smoker, n (%)	10 (12.82)	16 (19.75)	46 (30.66)	13 (16.46)	47 (31.97)	2 (40.00)
Age (year)	44.79±16.30	40.10±15.47	47.43±11.72	40.19±15.00	47.15±12.25	51.40±9.84
Duration (m)	NA	23.31±43.75	20.23±48.75	19.53±31.27	22.85±54.23	4.20±1.10
TSH (mIU/L)	NA	1.61±4.87	3.59±8.98	4.00±10.32	2.09±4.40	6.79±8.53
TSAb (IU/L)	NA	13.85±14.76	16.26±14.06	13.80±14.63	15.07±15.25	11.61±13.55
FT4 (pmol/L)	NA	16.53±11.39	16.34±12.66	16.54±12.06	16.35±10.82	11.83±1.29
ULR						
≥2mm, n (%)	NA	24 (29.63)	126 (84.00)	6 (7.59)	139 (94.56)	5 (100.00)
<2mm, n (%)	NA	57 (70.37)	24 (16.00)	73 (92.41)	8 (5.44)	0 (0.00)
Diplopia						
Yes, n (%)	0 (0.00)	29 (35.80)	107 (71.33)	29 (36.71)	103 (70.07)	5 (100.00)
No, n (%)	78 (100.00)	52 (64.20)	43 (28.67)	50 (63.29)	44 (29.93)	0 (0.00)
CAS	NA	1.00 (1.00, 2.00)	4.00 (3.00, 5.00)	2.00 (1.00, 2.00)	4.00 (3.00, 5.00)	4.00 (4.00, 5.00)
Treatment						
Previous medications, n (%)	0 (0.00)	18 (22.22)	52 (34.67)	11 (13.92)	58 (39.46)	5 (100.00)
None, n (%)	78 (100.00)	63 (77.78)	98 (65.33)	68 (86.08)	89 (60.54)	0 (0.00)
Exophthalmos by Hertel (mm)	NA	18.01±3.00	18.40±3.88	17.44±3.18	18.71±3.76	18.2±3.03
LPS_T (mm)	1.24 (1.06,1.40)	1.68 (1.46,2.10)	2.18 (1.75,3.03)	1.65 (1.40,2.04)	2.18 (1.78,3.05)	3.17 (2.64,3.68)
LPS_SIR	0.93 (0.80,1.04)	1.03 (0.88,1.13)	1.31 (1.10,1.86)	1.03 (0.89,1.16)	1.30 (1.09,1.85)	1.94 (1.64,2.47)

Data are means ± SD or median (interquartile ranges) or number (percentage) for indicated number of patients in each group.

TSH, thyroid-stimulating hormone; TSAb, thyrotropin receptor-stimulating antibody; FT4, free thyroxine; ULR, upper lid retraction; LPS_T, the maximum thickness of the levator palpebrae superioris muscle; LPS_SIR, the signal intensity ratio between the muscle and ipsilateral brain white matter.

Based on CAS, TAO eyes were defined as active phase if CAS is ≥ 3/7 and inactive phase otherwise. Based on the EUGOGO classification of severity, TAO eyes were divided into mild, moderate-to-severe, and sight-threatening.

### Grouping situation

According to the 2021 clinical practice guidelines for the medical management of TAO proposed by EUGOGO (7), the study subjects underwent evaluation using CAS and the EUGOGO classification of severity. Based on CAS, TAO eyes were classified as active phase if CAS is ≥3/7 and inactive phase otherwise. Based on the EUGOGO classification of severity, TAO eyes were divided into mild, moderate-to-severe, and sight-threatening.

### MRI acquisition

Subjects underwent orbital examination with the 3-Tesla Skyra MRI System (Siemens Healthcare, Erlangen, Germany) within 7 days of the ophthalmic examination. During the scan, the patient was in a supine position, the head was placed in the head coil, eyes were closed and focused on the front, and eye movements were restricted. A sagittal acquisition coincided with the longitudinal axis of the coil. Coronal Dixon-T2WI was performed with the following imaging parameters: repetition time (TR) 4,000 ms, echo time (TE) 91 ms, field of view (FOV) 15 cm×15 cm, matrix 320×320, slice thickness 3 mm, and slice spacing 0.6 mm; the images were sent to the PACS system.

### Image processing

**Figure 1:**
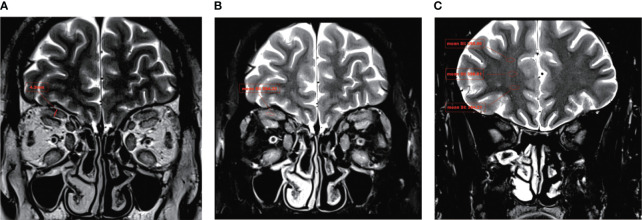
A typical Dixon-T2WI MRI in TAO. In a patient with moderate-to-severe TAO, the T2WI sequence revealed a significant thickening of the LPS and a notable increase in signal intensity. **(A)** The maximum thickness of the LPS muscle (LPS_T) was measured on coronal in-phase Dixon-T2WI images. **(B)** The maximum signal intensity of the LPS muscle (LPS_SI) was measured on coronal water-phase Dixon-T2WI images. **(C)** The signal intensity in ipsilateral brain white matter was measured on coronal water-phase Dixon-T2WI images.

Image analysis was conducted by two experienced radiologists using a single-blind method. LPS_T was measured on the coronal in-phase Dixon-T2WI image (**Figure 1A**). The maximum signal intensity of LPS was measured on the coronal water-phase Dixon-T2WI image (**Figure 1B**). The signal intensity in ipsilateral brain white matter regions of interest was measured three times on coronal water-phase Dixon-T2WI images showing the maximum white matter area of each brain, and an average was taken (**Figure 1C**). The ratio of the maximum signal intensity of LPS to the mean signal intensity of brain white matter (LPS_SIR) was calculated (27, 28).

### Statistical analysis

R4.22 was used for statistical analysis. The intraclass correlation coefficient (ICC) was used to test the consistency and repeatability of the two observers. Measurement data with normal distributions are represented by mean ± standard deviation, and those with skewed distributions are represented by median (interquartile range). Normally distributed data were analyzed by *t*-test or analysis of variance (ANOVA), and non-normally distributed data were analyzed by nonparametric Kruskal–Wallis *H*-test or Wilcoxon signed-rank test. The Pearson correlation coefficient (*r*) was used to describe correlation between two samples with normal distributions, and the Spearman correlation coefficient was used for correlations of non-normally distributed data. Diagnostic efficacy was assessed using the area under the receiver operating characteristic (ROC) curve (AUC). The Youden index determined the best cutoff point for ROC analyses. *p* < 0.05 was considered statistically significant.

## Results

### Clinical characteristics

**Figure 2:**
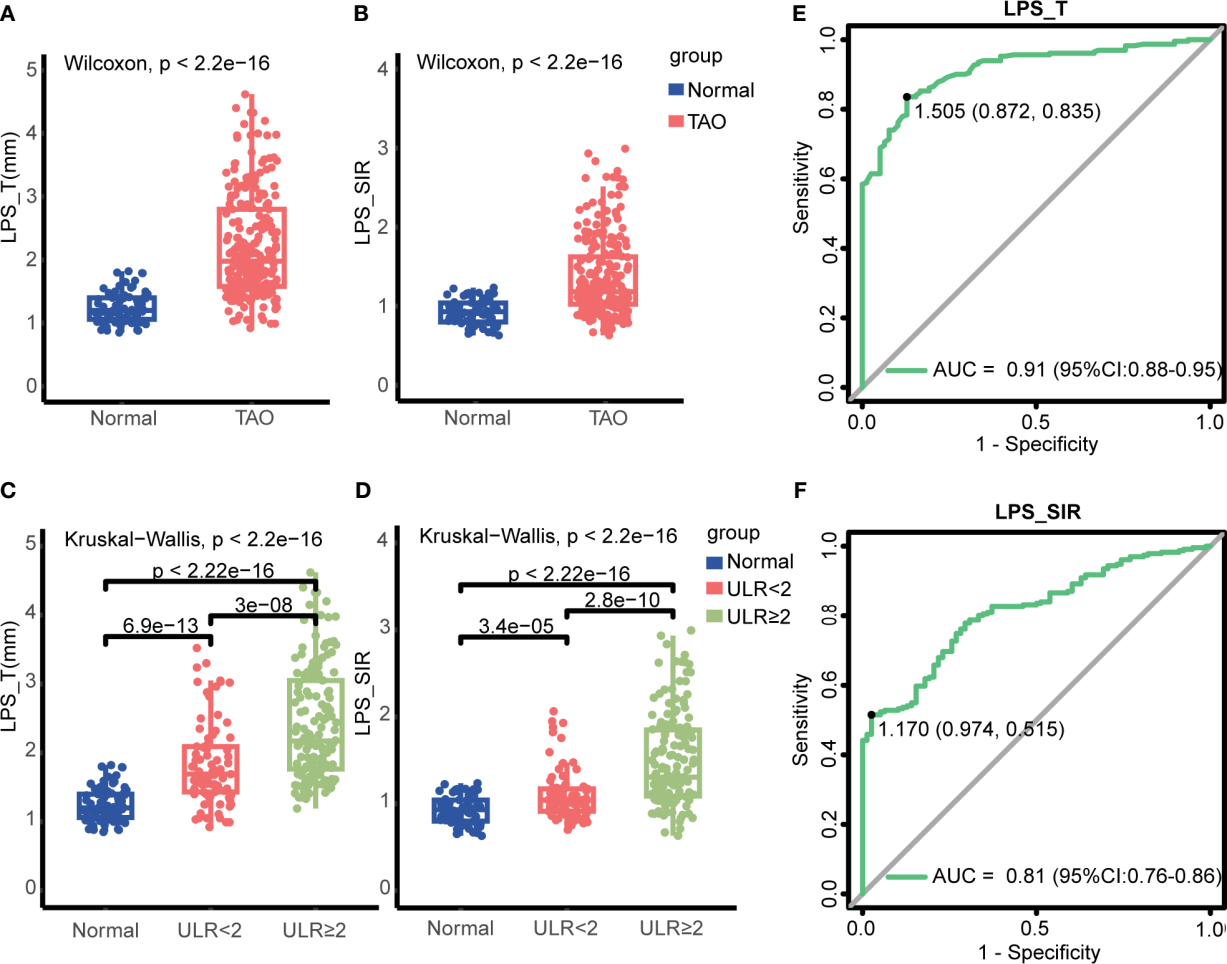
LPS_T and LPS_SIR in the differences between different TAO groups and diagnosis efficiency. **(A, B)** Comparison of the TAO and normal groups. **(C, D)** Comparison among the normal, ULR < 2 mm, and ULR ≥ 2 mm groups. LPS_T = the maximum thickness of the levator palpebrae superioris muscle. LPS_SIR = the signal intensity ratio between the muscle and ipsilateral brain white matter. **(E)** ROC for LPS_T to diagnose TAO, with a critical value of 1.505 mm. **(F)** ROC for LPS_SIR to diagnose TAO, with a critical value of 1.170.

This is a retrospective study. The TAO group consisted of 231 eyes (119 patients, 52.38% men; average age, 44.79 ± 16.30 years), while the normal group included 78 eyes (39 volunteers, 48.72% men; average age, 44.79 ± 16.30 years). The two experienced radiologists showed high consistency in measuring LPS_T and LPS_SIR, with an ICC of ≥0.90 for both. Statistical analysis revealed no significant difference in age and gender between the TAO and normal groups (*p* > 0.05). However, the smoking history in the TAO group was higher than that in the normal group (*p* < 0.05). There were statistically significant differences in LPS_T [2.21 (1.58, 2.80) mm vs. 1.24 (1.06, 1.40) mm, *p* < 2.2e-16] and LPS_SIR [1.18 (1.02, 1.63) vs. 0.93 (0.80, 1.04), p < 2.2e-16] between the TAO and normal groups. In the TAO group, LPS_T (*p* = 3e-08) and LPS_SIR (*p* = 2.8e-10) in eyes of patients with ULR ≥ 2 mm were also significantly higher than those with ULR < 2 mm (**Figures 2A–D**; **Table 1**). LPS_T and LPS_SIR were analyzed using ROC. When an LPS_T cutoff of 1.505 mm was applied, it achieved the best diagnostic performance for TAO, with a sensitivity of 83.5%, a specificity of 87.2%, and an AUC of 0.91 (*p* = 3.03e-28, **Figure 2E**). Applying an LPS_SIR cutoff of 1.170 resulted in a sensitivity of 51.5%, a specificity of 97.4%, and an AUC of 0.81 (*p* = 1.05e-16, **Figure 2F**).

### Correlations with disease activity and severity

In the study, based on CAS, TAO eyes were classified as active phase (64.94%) and inactive phase (35.06%). Based on the EUGOGO classification of severity, TAO patients were divided into the mild (34.20%), moderate-to-severe (63.64%), and sight-threatening (2.16%) groups. CAS in the active phase was higher than that in the inactive phase [4.00 (3.00, 5.00) vs. 1.00 (1.00, 2.00), *p* < 0.05]. CAS in the moderate-to-severe group was higher than that in the mild group [4.00 (3.00, 5.00) vs. 2.00 (1.00, 2.00), *p* < 0.05]. There was no statistically significant difference in CAS between the moderate-to-severe and the sight-threatening group (*p* > 0.05). There were no significant differences in exophthalmos between the active phase and the inactive phase (*p* > 0.05). However, the degree of ULR (84.00% vs. 29.63%), the history of diplopia (71.33% vs. 35.80%), and the history of previous medications (34.67% vs. 22.22%) in the active phase were higher than those in the inactive phase (*p* < 0.05). There was no statistically significant difference in exophthalmos between the EUGOGO group (*p* > 0.05). There were significant differences in the degree of ULR (7.59% vs. 94.56% vs. 100.00%), the history of diplopia (36.71% vs. 70.07% vs. 100.00%), and the history of previous medications (13.92% vs. 39.46% vs. 100.00%) among the mild, moderate-to-severe, and sight-threatening groups (*p* < 0.05) (**Table 1**).

**Figure 3:**
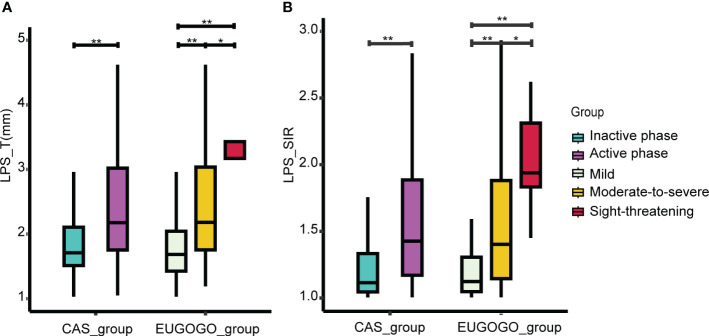
Comparison of LPS_T and LPS_SIR between groups. **(A)** Comparison of LPS_T between different groups. **(B)** The comparison of LPS_SIR between different groups. *p < 0.05, **p < 0.001.

**Figure 4:**
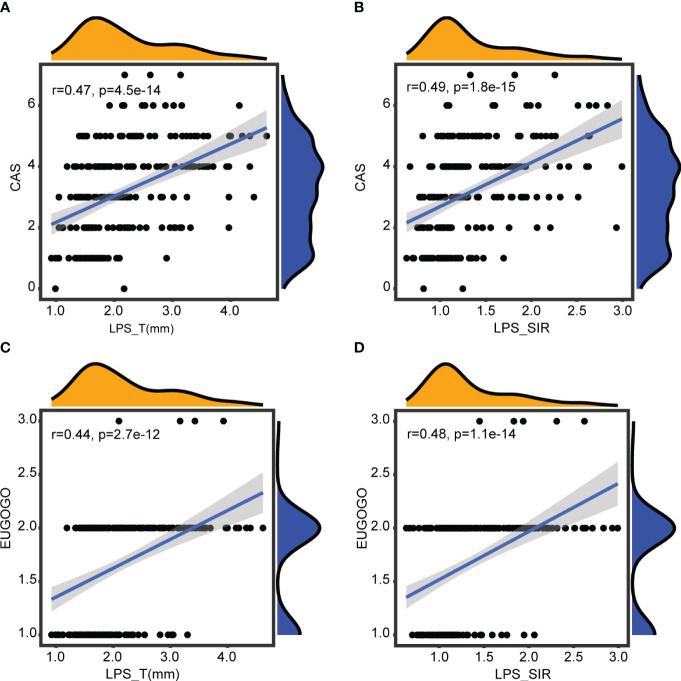
Correlations with disease activity and severity. **(A)** Correlation between LPS_T and CAS. **(B)** Correlation between LPS_SIR and CAS. **(C)** Correlation between LPS_T and EUGOGO of severity. **(D)** Correlation between LPS_SIR and EUGOGO of severity.

There were no significant differences in patient clinical information between CAS groups or EUGOGO classification of severity groups. However, statistically significant differences in both LPS_T and LPS_SIR were identified within the active phase and the inactive phase (*p*_LPS_T_ = 2.8e-07, *p*_LPS_SIR_ = 3.9e-12). Similarly, there were statistically significant differences in these indicators among the mild, moderate-to-severe, and sight-threatening groups (*p*_LPS_T_ = 2e-10, *p*_LPS_SIR_ = 2.8e-12, **Figures 3A, B**). Spearman correlation analysis showed that LPS_T and LPS_SIR were significantly positively correlated with disease activity (*R*_LPS_T_ = 0.47, *p*_LPS_T_ = 4.5e-14, *R*_LPS_SIR_ = 0.49, *p*_LPS_SIR_ = 1.8e-15, **Figures 4A, B**). Correlation with disease severity was positive for these variables (*R*_LPS_T_ = 0.44, *p*_LPS_T_ = 2.7e-12, *R*_LPS_SIR_ = 0.48, *p*_LPS_SIR_ = 1.1e-14, **Figures 4C, D**).

### LPS_T and LPS_SIR in the assessment of disease activity and severity

**Figure 5:**
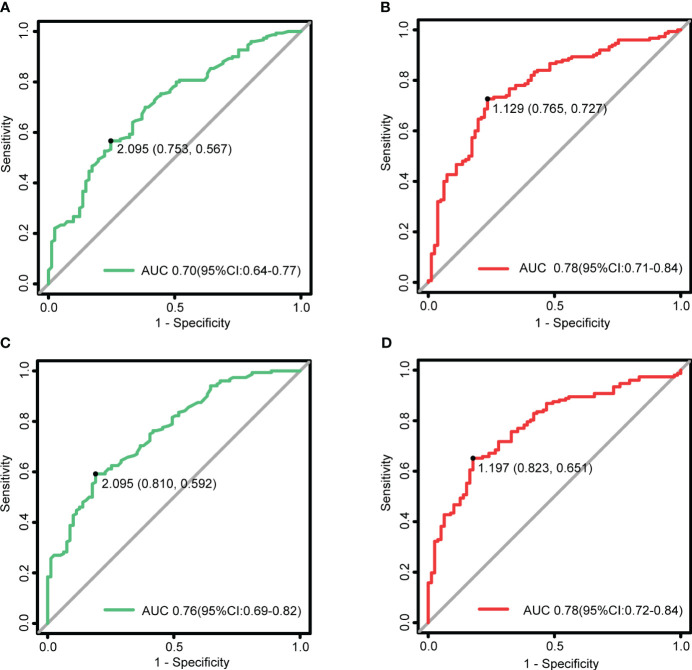
LPS_T and LPS_SIR in the assessment of disease activity and severity. **(A)** ROC for LPS_T to diagnose the active TAO, with a critical value of 2.095 mm. **(B)** ROC for LPS_SIR to diagnose the active TAO, with a critical value of 1.129. **(C)** ROC for LPS_T to judge the moderate-to-severe and above, with a critical value of 2.095 mm. **(D)** ROC for LPS_SIR to judge the moderate-to-severe and above, with a critical value of 1.197.

LPS_T and LPS_SIR underwent ROC analysis. As shown in **Figure 5**, when an LPS_T cutoff of 2.095 mm was used for judging the active TAO, the sensitivity was 56.7%, the specificity was 75.3%, and the AUC was 0.70 (*p* = 1.40e-07). An LPS_SIR cutoff of 1.129 resulted in a sensitivity of 72.7%, a specificity of 76.5%, and an AUC of 0.78 (*p* = 1.93e-12).

When an LPS_T cutoff of 2.095 mm was used for judging the moderate-to-severe and above, the sensitivity was 59.2%, the specificity was 81.0%, and the AUC was 0.76 (*p* = 6.92e-11). An LPS_SIR cutoff of 1.197 resulted in a sensitivity of 65.1%, a specificity of 82.3%, and an AUC of 0.78 (*p* = 1.19e-12).

## Discussion

In this study, we provided a clinical study with 309 eyes (231 TAO eyes and 78 normal eyes) to assess the diagnostic value of LPS_T and LPS_SIR in TAO. The results illustrate that both indicators exhibit diagnostic performance, underscoring their significance in TAO evaluation. Furthermore, distinguishing itself from existing domestic and international studies, this study uniquely evaluated TAO’s activity and severity using measurements of LPS_T and LPS_SIR derived from Dixon-T2WI sequences. The results indicate that this method is straightforward, quantitative, highly accurate in assessment, and provides intergroup cutoff. This implies that LPS_T and LPS_SIR can serve as objective imaging indicators to assist in the diagnosis of TAO. Moreover, they can also serve as objective quantitative indicators to assist in assessing the condition, providing evidence for further exploration of imaging criteria to evaluate and predict treatment efficacy.

In this study, LPS_SIR represents the degree of inflammatory edema in the LPS, while LPS_T signifies the extent of its swelling (29). The results indicated that both LPS_T and LPS_SIR were significantly higher in the TAO group compared to the normal group, which is consistent with the findings of Duan et al (25). Furthermore, among TAO eyes, those with ULR ≥ 2 mm exhibited significantly higher values of LPS_T and LPS_SIR compared to those with ULR < 2 mm. This suggests that these two features can reflect the condition of eyelid retraction and provide imaging support for the diagnosis and treatment of TAO. We proposed a cutoff for LPS_T and LPS_SIR to diagnose TAO. We found that a cutoff of 1.505 mm for LPS_T yielded the highest sensitivity and specificity for TAO diagnosis. Likewise, for LPS_SIR, the identified cutoff was 1.170, also indicating the highest sensitivity and specificity. Therefore, LPS_T and LPS_SIR have excellent diagnostic value and are important indicators in diagnosing TAO.

Our research findings further validate the positive correlations between LPS_T and LPS_SIR with CAS and the EUGOGO classification of severity. Moreover, both of these features exhibit significant increases in the eyes of patients with active and moderate-to-severe TAO, thereby showing the diagnostic capability of the Dixon-T2WI sequence in assessing orbital inflammation. Based on ROC analysis, we found that both LPS_T and LPS_SIR demonstrated good performance for assessing the activity and severity of TAO. Among them, LPS_SIR exhibited the best diagnostic performance with the highest AUC value. Thus, we propose a cutoff of 1.129 for LPS_SIR to diagnose the active TAO. Similarly, a cutoff value of 1.197 for LPS_SIR was proposed to diagnose the moderate-to-severe and above. However, the diagnostic performance of LPS_T appears to have acceptable sensitivity but low specificity. The following are the possible reasons for this: (1) insufficient sample size may lead to biased errors; (2) assessment of TAO based solely on MRI features of LPS may not provide a comprehensive evaluation and would benefit from incorporating multiple variables; and (3) the use of CAS and the EUGOGO classification of severity as indicators for disease assessment in determining the cutoff using ROC analysis, which is subjective and has insufficient sensitivity. This also suggests that using CAS and the EUGOGO classification of severity alone may not fully reflect the condition of TAO.

This study has limitations. Firstly, it is a retrospective, single-center study, which introduces inherent bias. Secondly, the sample size of the sight-threatening group was relatively small, which may not be representative of the whole population. Additionally, TAO is a complex disease involving multiple factors, and its clinical manifestations are multiple factors. It is difficult to comprehensively evaluate TAO based on MRI features of LPS alone, and it is necessary to combine multiple objective indicators to evaluate TAO clinically. Finally, in the future, a larger prospective cohort should be conducted to further validate the quantitative value of the LPS thickness and SIR in clinical practice.

## Conclusions

In conclusion, the maximum thickness and SIR of LPS can serve as objective indicators to assist in the diagnosis and quantitative evaluation of TAO, alleviating the limitations of CAS and EUGOGO classifications, which suffer from pronounced subjectivity and limited comparability. These indicators provide a research basis for exploring imaging criteria in the next step for evaluating and predicting efficacy.

## Data availability statement

The raw data supporting the conclusions of this article will be made available by the authors, without undue reservation.

## Ethics statement

The studies involving humans were approved by the ethical review boards of Shunde Hospital of Southern Medical University (KYLS20220723). The studies were conducted in accordance with the local legislation and institutional requirements. Written informed consent for participation was not required from the participants or the participants' legal guardians/next of kin in accordance with the national legislation and institutional requirements

## Author contributions

DL: Conceptualization, Data curation, Formal analysis, Investigation, Methodology, Software, Validation, Visualization, Writing – original draft. YD: Conceptualization, Data curation, Formal analysis, Investigation, Methodology, Writing – original draft. KH: Data curation, Formal analysis, Software, Investigation, Writing – original draft. CS: Data curation, Software, Methodology, Investigation, Writing – original draft. YO: Data curation, Formal analysis, Investigation, Writing – original draft. XL: Data curation, Methodology, Investigation, Writing – original draft. JS: Conceptualization, Supervision, Writing – review & editing. HC: Funding acquisition, Project administration, Resources, Supervision, Writing – review & editing. All authors contributed to the article and approved the submitted version.

## Supplementary material

Supplementary Figure 1 Patient selection and MRI evaluation flowchart np: the number of patients. ne: the number of affected eyes. TAO: thyroid-associated ophthalmopathy. CAS: Clinical Activity Score. EUGOGO: European Group on Graves’ orbitopathy.
