# Supplementary figures and images for: Assessing the relationship between levator palpebrae superioris and thyroid-associated ophthalmopathy using the Dixon-T2WI sequence
Source: Front Endocrinol (Lausanne). 2024 May 28;15:1387217. doi: 10.3389/fendo.2024.1387217 (PMC11168108; doi:10.3389/fendo.2024.1387217)

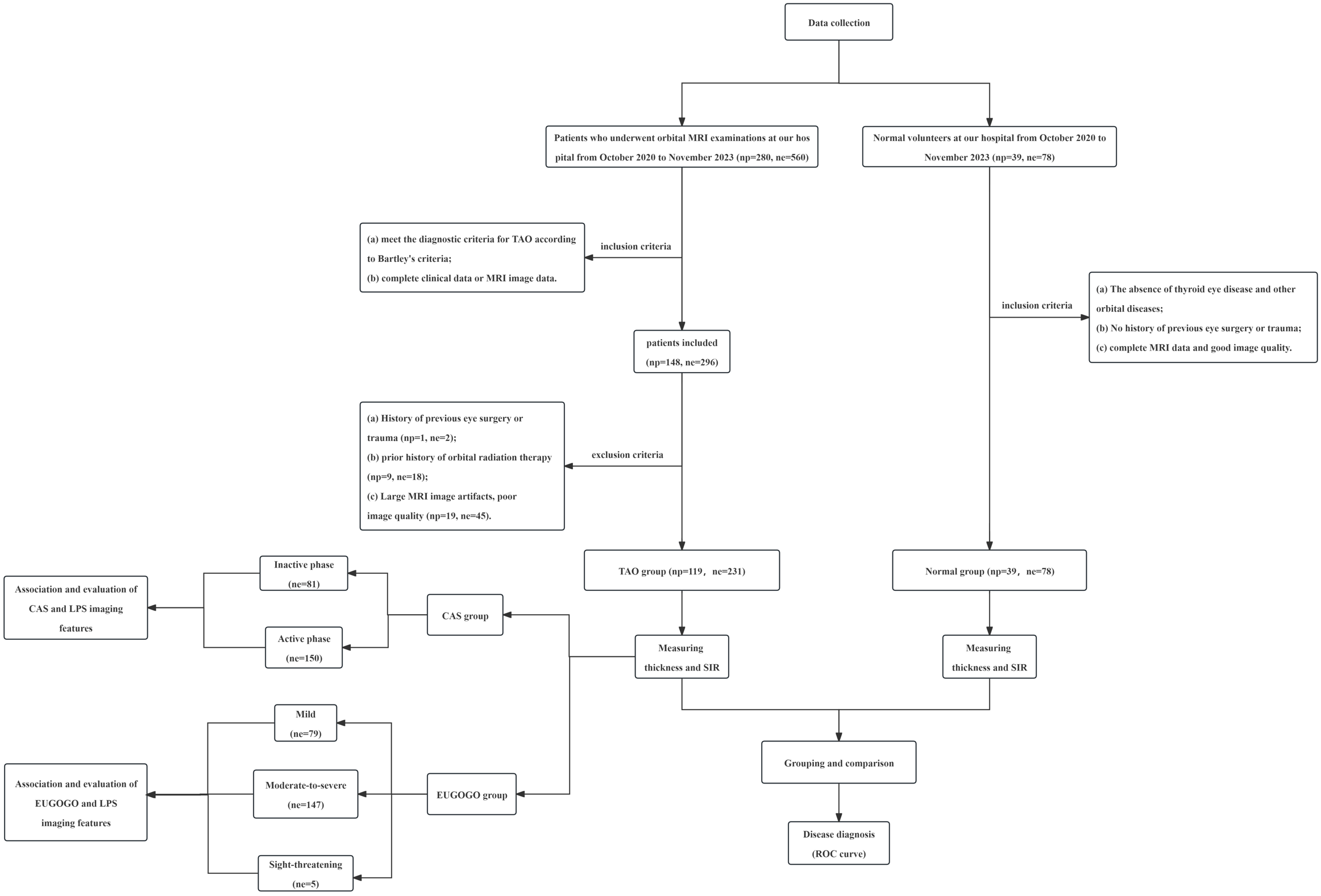

Supplement: Supplementary Figure 1 — Patient selection and MRI evaluation flowchart np: the number of patients. ne: the number of affected eyes. TAO: thyroid-associated ophthalmopathy. CAS: Clinical Activity Score. EUGOGO: European Group on Graves’ orbitopathy. [file Image_1.pdf]
